# Genetically Different Isolates of the Arbuscular Mycorrhizal Fungus *Rhizophagus irregularis* Induce Differential Responses to Stress in Cassava

**DOI:** 10.3389/fpls.2020.596929

**Published:** 2020-12-02

**Authors:** Ricardo Peña, Chanz Robbins, Joaquim Cruz Corella, Moses Thuita, Cargele Masso, Bernard Vanlauwe, Constant Signarbieux, Alia Rodriguez, Ian R. Sanders

**Affiliations:** ^1^Department of Ecology and Evolution, University of Lausanne, Lausanne, Switzerland; ^2^International Institute for Tropical Agriculture (IITA) Kenya, Nairobi, Kenya; ^3^International Institute for Tropical Agriculture (IITA) Cameroon, Yaoundé, Cameroon; ^4^Laboratory of Ecological Systems (ECOS), Swiss Federal Institute of Technology Lausanne (EPFL), Lausanne, Switzerland; ^5^Department of Biology, National University of Colombia, Bogotá, Colombia

**Keywords:** cassava, Africa, intraspecific genetic variability, *Rhizophagus irregularis*, drought recovery, Water-stress

## Abstract

Water scarcity negatively impacts global crop yields and climate change is expected to greatly increase the severity of future droughts. The use of arbuscular mycorrhizal fungi (AMF) can potentially mitigate the effects of water stress in plants. Cassava is a crop that feeds approximately 800 million people daily. Genetically different isolates of the AMF *R. irregularis* as well as their clonal progeny have both been shown to greatly alter cassava growth in field conditions. Given that cassava experiences seasonal drought in many of the regions in which it is cultivated, we evaluated whether intraspecific variation in *R. irregularis* differentially alters physiological responses of cassava to water stress. In a first experiment, conducted in field conditions in Western Kenya, cassava was inoculated with two genetically different *R. irregularis* isolates and their clonal progeny. All cassava plants exhibited physiological signs of stress during the dry period, but the largest differences occurred among plants inoculated with clonal progeny of each of the two parental fungal isolates. Because drought had not been experimentally manipulated in the field, we conducted a second experiment in the greenhouse where cassava was inoculated with two genetically different *R. irregularis* isolates and subjected to drought, followed by re-watering, to allow recovery. Physiological stress responses of cassava to drought differed significantly between plants inoculated with the two different fungi. However, plants that experienced higher drought stress also recovered at a faster rate following re-watering. We conclude that intraspecific genetic variability in AMF significantly influences cassava physiological responses during water stress. This highlights the potential of using naturally existing variation in AMF to improve cassava tolerance undergoing water stress. However, the fact that clonal progeny of an AMF isolate can differentially affect how cassava copes with natural drought stress in field conditions, highlights the necessity to understand additional factors, beyond genetic variation, which can account for such large differences in cassava responses to drought.

## Introduction

Water scarcity negatively impacts crop yields at a global scale, reducing average yields by more than 50% (Wang et al., 2003). Climate change is expected to increase the severity of drought (FAO, 2012; IPCC, 2018), thus, exacerbating the threat of global yield reductions. This, combined with a growing world population, threatens the food security of developing nations and small-holder farmers. Practical solutions to circumvent this threat are needed to ensure food security in a changing planet.

Drought disrupts the photosynthetic apparatus, negatively affecting productivity. Stomatal closure is a common drought stress response, but non-stomatal limitation to photosynthesis occurs under more severe drought (Flexas et al., 2004; Grassi and Magnani, 2005; Signarbieux and Feller, 2011). This occurs when absorbed light exceeds the amount required for CO_2_ assimilation, causing an energy imbalance in photosystem II (PSII) (Müller et al., 2001; De Ronde et al., 2004). Because of this, chlorophyll *a* (Chl *a*) fluorescence is a non-invasive, quantitative measurement that indicates plant stress levels, providing information about PSII efficiency during stress conditions (Maxwell and Johnson, 2000; Strasser et al., 2004; Niu and Rodriguez, 2008; Signarbieux and Feller, 2011; Zushi and Matsuzoe, 2017). Indeed, this measurement is commonly used when phenotyping crop varieties for drought tolerance and for evaluating the impact of mycorrhizal fungi inoculation on plants (Tsimilli-Michael and Strasser, 2008; Czyczyło-Mysza et al., 2013; Kalaji et al., 2016).

Cassava (*Manihot esculenta* Cranz) is a vital food crop in the tropics and sub-tropics, including sub-Saharan Africa, where it is cultivated as a subsistence crop. Cassava is a staple food to an estimated 800 million people worldwide (FAO, 2013). There is a lot of interest in cassava as a food security crop, because during environmental perturbation, particularly drought, cassava yields are relatively stable compared to cereals (El-Sharkawy, 2006, 2014). Although cassava is known to be relatively drought tolerant, reduced water availability can still reduce yields far below the maximum potential (Oyetunji et al., 2007). In order to supply food in areas of poor food security in Africa, cassava is increasingly being grown on marginal lands where plants suffer water deficit almost annually (El-Sharkawy, 2006; FAO, 2013). Ekanayake et al. (2004) showed that a decrease in cassava chlorophyll production during water stress has a negative effect on PSII efficiency of cassava, and consequently, affects root yield negatively. Therefore, monitoring changes in Chl *a* fluorescence kinetics in cassava can provide detailed information on the functioning of its photosynthetic apparatus and detect when the system is under stress.

It has been demonstrated that the use of AMF can help to mitigate the effects of drought stress on plants (Augé, 2001; Oyetunji et al., 2007; Hu et al., 2017). AMF form a symbiotic association with roots of most crop species, including cassava. In this association, AMF obtain mineral nutrients, particularly phosphate, from the soil and transport them to plant roots. In return, AMF obtain carbohydrates and lipids from plant roots (Harrison, 1999; Keymer et al., 2017). However, these fungi can also protect plants from pathogens and improve water stress tolerance (St-Arnaud and Vujanovic, 2006; Oyetunji et al., 2007; Tsimilli-Michael and Strasser, 2008; Boldt et al., 2011; García-Sánchez et al., 2014; Séry et al., 2016) although the mechanism by which AMF improve drought tolerance is poorly understood.

*Rhizophagus irregularis* is a model AMF species commonly found in soils worldwide (Savary et al., 2018) and has a great potential to improve the productivity of cassava (Ceballos et al., 2013, 2019; Rodriguez and Sanders, 2015; Aliyu et al., 2019). Furthermore, this AMF species can be cultivated *in-vitro* and can be efficiently produced on a large-scale, making it a promising candidate for inoculation of cassava. Additionally, genetic variation in this fungus has been extensively studied (Wyss et al., 2016; Chen et al., 2018; Savary et al., 2018; Masclaux et al., 2019), and shown to significantly alter plant growth (Munkvold et al., 2004; Koch et al., 2006, 2017). However, clonally produced *R. irregularis* siblings of a given *R. irregularis* isolate also display very large differences in fungal quantitative growth traits and plant growth, for example, in rice and cassava (Angelard et al., 2010; Ehinger et al., 2012; Ceballos et al., 2019). *R. irregularis* isolates exist as dikaryons, which means that they contain a population of nuclei with two distinct genotypes, or as homokaryons where all nuclei are of one identical genotype. Clonally grown offspring of both dikaryon and homokaryon isolates induce very large differences in cassava growth in conventional farming conditions in Colombia, Kenya and Tanzania (Ceballos et al., 2019). The role of variation in AMF in influencing drought stress responses of cassava remains poorly investigated.

Given that both among and within-isolate genetic variation of *R. irregularis* isolates causes large differences in cassava growth, such variation could also potentially influence cassava tolerance to drought stress. Therefore, we hypothesized that among and within isolate variation in *R. irregularis* are not only drivers of cassava productivity, but also of cassava physiological stress responses to water deficit conditions.

**FIGURE 1:**
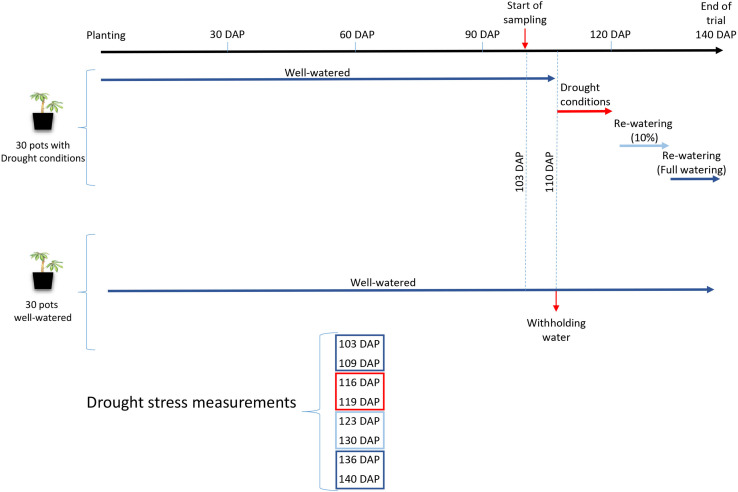
Schematic timeline of the design of Experiment 2, showing the watering regimes. Colors dark blue, red and light blue represent the different watering regimes imposed during the experiment.

In order to address this hypothesis, two experiments were performed to assess cassava photosynthetic responses under water stress when inoculated with different *R. irregularis* lines. The first experiment was conducted under field conditions in Western Kenya, where cassava regularly experiences natural drought. Cassava was not experimentally subjected to drought but the response was measured when plants were exposed to naturally occurring drought conditions during the dry season in Kenya. The goal of this experiment was to measure how single spore sibling *R. irregularis* cultures (hereafter called progeny) from homokaryon and dikaryon parents influenced cassava stress responses. We observed that cassava stress levels during the dry season were indeed influenced by the identity of the AMF inoculant. Because the plants had not been experimentally subjected to a drought treatment, we performed a second experiment where we subjected cassava, inoculated with two genetically different *R. irregularis* isolates, to a drought treatment followed by a recovery period (Figure 1). The goal of this second experiment was to test whether genetically distinct *R. irregularis* isolates induce differential physiological responses in cassava during drought stress and a subsequent recovery period.

## Materials and Methods

### Experiment 1. Field Experiment (Kenya)

#### Location of the Experiment

The field experiment was established in Ukwala-Kawayo in Western Kenya (00° 15’ 12.1” N and 34° 10’ 32.7” E), with an average annual temperature of 22.3°C, 75% relative humidity and an annual precipitation of 1468 mm. The soil was composed of 12% sand, 72% clay and 16% silt with a pH(H_2_O) of 3.94, organic carbon 0.65 g kg^–1^, total N 0.08 g kg^–1^, available P 6 mg kg^–1^, exchangeable K 0.09 cmol_(__+__)_ kg^–1^, exchangeable Ca 0.51 cmol_(__+__)_ kg^–1^ and exchangeable Mg 2.33 cmol_(__+__)_ kg^–1^.

#### Plant and Fungal Material

A local cassava variety (Fumba Chai) that represents the most commonly cultivated variety in the region was used. Parental isolates (C2 and C3) originated from the Hausweid field station, at Tänikon, Switzerland (Koch et al., 2004), where they have been maintained in identical conditions at the University of Lausanne *in vitro* since 2000. Single-spore cultures, also known as single spore lines (SSLs), were raised *in-vitro* at the University of Lausanne, and originated from parental isolates (C2 and C3) (Ceballos et al., 2019; Supplementary Table S1) in exactly the same conditions as parental isolates. All cultures were transferred to Symbiom s.r.o. (Lanskroun, Czech Republic) for production at a larger scale required for the field experiment. To do this, each parental and SSL of *R. irregularis* was produced in an identical *in vitro* culture system by Symbiom s.r.o. (Lanskroun, Czech Republic). To avoid potential batch effects each parental and SSL was produced in 8 separate culture units and the fungal material (spores and hyphae) produced in each unit for each isolate or SSL was pooled and mixed. Spore density was then counted so that each culture could be mixed with a sterile inert carrier (calcified diatomite) to a standardized spore density of 1000 spores g^–1^.

#### Isolate Selection

The parental isolates C2 (a homokaryon) and C3 (a dikaryon) were selected based on genetic differences observed between them in previous studies (Wyss et al., 2016; Savary et al., 2018). Isolates C2 and C5 were shown to be genetically indistinguishable. SSLs of parental isolates (C2 and C3) were each initiated from a single spore of the parental isolate (Ceballos et al., 2019). Each spore contains multiple nuclei originating from the parental isolate. Therefore, each SSL originating from a homokaryon parent should be genetically identical. However, offspring from a dikaryon parent can be genetically variable due to the inheritance of a disproportionate number of the two nucleus genotypes (Masclaux et al., 2018).

#### Design and Establishment of the Field Experiment

The experiment was set up as a randomized complete block design comprising 15 inoculation treatments distributed in 10 blocks, with one replicate of each treatment per block. A total of 13 *R. irregularis* isolates and SSLs were used, including 3 parental isolates (C2, C3 and C5) and 10 SSLs (Supplementary Table S1). In addition, there were 2 control treatments, either adding only the sterile carrier (inert substrate) with no fungus (Ca) or no addition (no fungus, no carrier) (No). The planting design comprised one plant of a given inoculation treatment surrounded by eight non-inoculated plants, in order to avoid possible cross contamination between treatments. This experiment covered an approximate area of 0.5 ha with a planting density of 10000 plants ha^–1^. The experiment was planted in June 2016 and harvested in April 2017.

Due to the low soil pH at the site, Minjingu Phosphate rock (12.8% total P and 38% CaO) was applied before planting at 460 kg ha^–1^, two weeks before planting, as a source of P and as a liming material (Szilas et al., 2008). Nitrogen and potassium fertilizers were applied at 25 days after planting (DAP). The amount of fertilizer applied was determined by the initial soil nutrient content, nutritional requirements and fertilizer use efficiency. The application rate was 150 kg N ha^–1^ and 180 kg K ha^–1^. The sources of nitrogen and potassium fertilizers were: urea and Muriate of potash (MOP). In all the treatments except the No and Ca control treatments, cassava stems (30 cm long) were inoculated with 1 g of the inoculum (containing 1000 spores), and this was placed around the stem of the cassava at planting.

#### Data Collection

We assessed cassava stress by measuring the polyphasic Chl *a* fluorescence transient (OJIP) (Strasser et al., 2004). We measured the Chl *a* fluorescence variables maximum quantum yield of photosystem II (PSII) F_v_/F_m_ (which is equivalent to TR_0_/ABS) and the performance index (PI) (Strasser et al., 2004; Tsimilli-Michael and Strasser, 2008). using a non-destructive method using a Handy PEA fluorometer (Plant Efficiency Analyser, Hansatech, Norfolk, United Kingdom). This was performed to evaluate potential damage caused to the photosystems by stress and to monitor physiological changes in the plant (Niu and Rodriguez, 2008; Zushi and Matsuzoe, 2017). While this apparatus measures a very large number of different variables, we focussed on the above variables because they are ones that have previously been demonstrated to be influenced by experimentally subjecting plants to water stress. Chl *a* fluorescence measurements were made on three fully expanded attached leaves per plant, each located in the mid-canopy of all replicate plants in all treatments. Before all measurements, leaves were dark-adapted for 20 min. Light intensity was 3000 μmol m^–2^ s^–1^, provided by an array of three high-intensity light-emitting diodes. The measurements were made at 120 (rooting phase), 210 (root thickening phase), and 300 DAP (dry matter accumulation phase).

We also measured soil moisture (SM) and the leaf water potential (LWP) at the same time points as the fluorescence measurements. The measurement at 210 DAP occurred in the low-rainfall season so as to measure naturally occurring drought stress (Supplementary Figure S1). LWP and SM were measured with a Scholander pressure chamber (Scholander et al., 1964) and a portable SM - ML3 ThetaProbe sensor (Delta-T^®^), respectively. Plant growth and AMF colonization data for this experiment are presented in Ceballos et al. (2019).

#### Statistical Analyses

All data were analyzed using the R statistical software (R Core Team, 2018; version 3.5.1) and JMP^®^ 13.2.0 (SAS institute Inc.). To test for significant differences among treatments, analysis of variance (ANOVA) was performed followed by a post-hoc Tukey honest significant difference (HSD) test. In this experiment, we first made repeated measures analysis on cassava stress variables throughout the sampling times (120, 210 and 300 DAP) to test for a sampling time effect. Second, data were then analyzed by separating the dataset in two groups: (1) plants inoculated with parental isolate C2 and its progeny, and (2) plants inoculated with parental isolate C3 and its progeny. We did this in order to test whether there was a differential effect on cassava among plants inoculated with a parental fungus and its progeny at each sampling time.

### Experiment 2. Greenhouse Experiment

#### Location of Experiment

This experiment was established in the greenhouse of the Department of Ecology and Evolution, University of Lausanne, Switzerland, under controlled conditions (temperature: 23–25 °C; relative humidity: ∼60%) in November 2017.

#### Plant and Fungal Material

Fresh stems of cassava variety CM4574-7 (CIAT, Colombia) were transported from a field site in Colombia in the last week of October 2017 and planted in pots in the greenhouse 1 week later. Variety CM4574-7 is well adapted to savanna conditions and is resistant to root rot and used for local consumption and industrial uses. *R. irregularis* parental lines A1 and C3 were produced in the same *in-vitro* culture system by Symbiom^®^ s.r.o. The parental lines A1 and C3 were chosen based on DNA sequence data showing these two isolates are genetically distinct (Wyss et al., 2016; Savary et al., 2018). The reason for using one of the isolates (A1) that was not used in the field experiment was because of inoculum availability and because this fungus was the subject of another study in the laboratory on transcriptional responses of plants and AMF to drought. However, the objective of this study was not to directly compare data between the first and the second experiment.

#### Experimental Design

The experiment was set up as a randomized design that comprised 3 inoculation treatments, 2 drought treatments and 10 replicates for each of the treatment combinations. Cassava plants were inoculated with either A1, C3 or a control comprised of inert sterile substrate with no fungus (Ca). A homogenized soil medium (sand : loam; 2:3) was prepared and steam sterilized (1 h). Sterilized soil was allowed to aerate for one week under a plastic cover to remove phytotoxic effects, before being used for planting (12 L pots). Inoculation was carried out 21 DAP. All plants were subjected to the same watering regime (90% of holding capacity, 35% volumetric SM) until 110 DAP. After 110 DAP, two different treatments were administered; with continued watering (watered) or by withholding water (drought). Water was withheld in the drought treatment until the leaves began dropping; a physiological response typical of cassava experiencing drought (Okogbenin et al., 2013). At 120 DAP, drought treated plants were re-watered and maintained at 10% of volumetric SM, until 130 DAP. From 131 DAP, drought treated plants received full water at 90% of holding capacity, until harvest (140 DAP) (Figure 1).

#### Data Collection

##### Non-destructive measurements

Plant height and main stem diameter were measured. Chl *a* fluorescence was measured as in Experiment 1. As Chl *a* fluorescence gives indications about the stress level of the plant related to the non-stomatal photosynthesis processes, it does not allow the detection of the direct effect on photosynthesis or how that stress is regulated at the stomatal level. We, therefore, measured photosynthetic capacity (A_max_) and stomatal conductance (g_smax_), i.e. at optimal conditions for light, temperature and vapor pressure deficit (VPD) allowing comparable measurements between treatments. A_max_ and g_smax_ were measured using an open path infrared gas analyser (IRGA) system connected to a 2.5 cm^2^ PLC-6 chamber (CIRAS-2, PP-Systems, Amesbury, United States) under optimal conditions for light (1200 μmol of photons m^–2^ s^–1^), at 25 ± 1°C, CO_2_ concentration of 400 ± 2 ppm, and maintaining a vapour pressure deficit (VPD) under 1.5 kPa in order to avoid any limitation on the stomatal opening. These measurements were made on the first fully expanded leaf of 6 replicate plants in all treatments. The same leaf of each plant was measured at each time point. In the case where a selected leaf fell due to drought, the next superior leaf was used for proceeding measurements. We also calculated the water use efficiency (WUE) as the ratio A_max_:g_smax_. All non-destructive measurements were made 8 times throughout the experiment at 103 DAP, 109 DAP, 116 DAP, 119 DAP, 123 DAP, 130 DAP, 136 DAP and 140 DAP, starting the week before withholding of water.

##### Destructive measurements

At 91 DAP, cassava roots were removed and stained following the method of Koske and Gemma (1989), except trypan blue was replaced by acid fuchsin 0.01%, and checked for AMF colonization using the grid-line intersection method (Giovanetti and Mosse, 1980). This was carried out to confirm that the roots of inoculated plants were colonized by AMF before inducing the drought. At harvest (140 DAP), plant material was separated into root biomass and or shoot biomass by removing all outgrowth from, but not including, the planted stake. After root biomass was measured, a randomized sample was preserved in 70 % ethanol. The percentage of root length colonized by AMF was then measured at a later date using the same method used previously.

Additionally, the following indices were calculated at the end of the trial:

Relative yield reduction (RYR) was estimated using the following equation:

$$R\,Y\,R=\left(\frac{Y\,w-Y\,d}{Y\,w}\right)$$

Where *Y*_*w*_ and *Y*_*d*_ are the means of root fresh weight (g plant^–1^) at harvest of each treatment under well-watered (*Y*_*w*_) and water-stressed conditions (*Y*_*d*_), respectively.

Drought susceptibility index (DSI) was determined using the following equation:

$$D\,S\,I=\frac{\left[1-\left(\frac{Y\,d_{i}}{Y\,w_{i}}\right)\right]}{D\,I\,I}$$

Where *Y_*d*__*i*_* and *Y_*w*__*i*_* are the means of root fresh weight (g plant^–1^) at harvest of individual treatments under water-stressed (*Y*_*d*_) and well-watered (*Y*_*w*_) conditions, respectively. DII is the drought intensity index (DII) which was calculated using the following equation:

$$D\,I\,I=1-\left(\frac{Y\,d}{Y\,w}\right)$$

Where *Y*_*d*_ and *Y*_*w*_ are the means of root fresh weight (g plant^–1^) at harvest of all treatments under water-stressed (*Y*_*d*_) and well-watered (*Y*_*w*_) conditions, respectively.

Recovery rate (RR) was elucidated using the following equation:

$$R\,R=\left({\left(\frac{f\,i}{s}\right)}^{\frac{1}{y}}-1\right)\,x\,100\%$$

Where *fi* is the mean of parameter at different sampling times during re-watering (123, 130, 136 and 140 DAP), *s* is the value of parameter at the maximum stage of drought (119 DAP) and *y* is the number of sampling times during re-watering.

RYR and DSI are widely used by plant breeders to assess drought tolerance when testing different plant genotypes or when phenotyping for improved traits for drought tolerance and here we used it to compare the DSI among AMF treatments (Grzesiak et al., 2013; De Oliveira et al., 2017; Peirone et al., 2018).

#### Statistical Analyses

All data were analyzed using the R statistical software (R Core Team, 2018; version 3.5.1) and JMP^®^ 13.2.0 (SAS institute Inc.). To test for significant differences among treatments, analysis of variance (ANOVA) was performed followed by a post-hoc Tukey honest significant difference (HSD) tests. First, we tested for a drought treatment effect on different cassava growth variables at the end of the trial. Second, we tested for an inoculation treatment effect on gas exchange and Chl *a* fluorescence at each sampling time. Third, we tested for an inoculation treatment effect on the RR of gas exchange and Chl *a* fluorescence of cassava during re-watering period and also on DSI at the end of the experiment in plants that had been subjected to drought. Additionally, we used Dunnett’s test to test for a drought treatment effect on gas exchange and Chl *a* fluorescence of cassava at each sampling time by taking watered plants as control.

## Results

### Experiment 1. Effect of Inoculation With Parental *R. irregularis* Isolates and Their SSL Progeny on Cassava Stress Responses Under Field Conditions

#### Chlorophyll *a* Fluorescence Analysis

**FIGURE 2:**
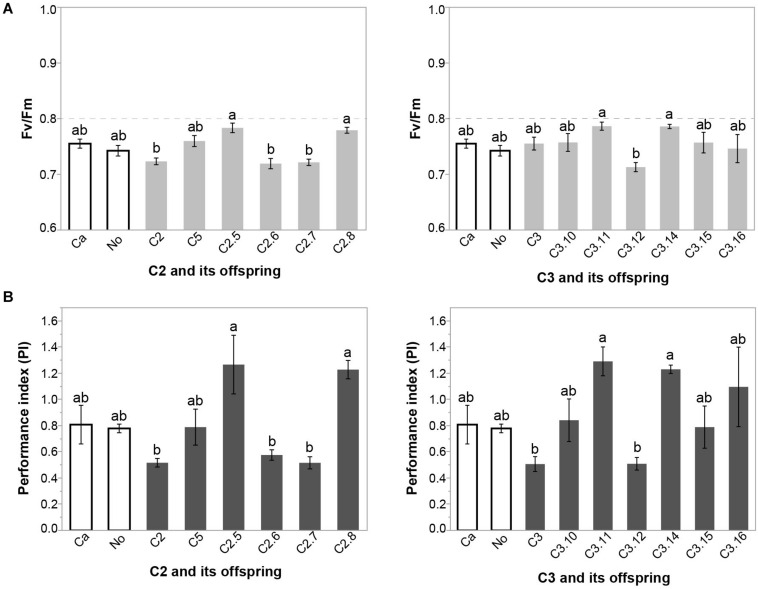
Effect of inoculation with parental isolates C2 and C3 and their SSL progeny on **(A)** maximum quantum yield of PSII (F_v_/F_m_) **(B)** performance index (PI) of cassava at 210 DAP in Experiment 1. Error bars represent ± S.E. Different letters above bars represent significant differences at *p* < 0.05 according to a Tukey post-hoc test.

At 210 DAP, the variables maximum quantum yield of PSII (F_v_/F_m_), the performance index (PI), leaf water potential (LWP) and soil moisture (SM) were all significantly lower than at 120 and 300 DAP (*p* < 0.01) (Table 1). There was no significant effect of inoculation on any of those variables at 120 and 300 DAP (Supplementary Tables S2–S3). However, F_v_/F_m_ and PI differed significantly among the inoculation treatments at 210 DAP (Supplementary Tables S2–S3). Plants inoculated with SSLs C2.5 and C2.8 exhibited a significantly higher F_v_/F_m_ ratio and PI than plants inoculated with their parent C2 or their siblings C2.6 and C2.7 at 210 DAP (Figure 2 and Supplementary Table S2), showing that variation in stress responses occurred among cassava plants inoculated with clonally produced mycorrhizal fungal siblings of a homokaryon parental fungus. Plants inoculated with SSL C3.11 and C3.14 had a higher F_v_/F_m_ than the sibling C3.12 at 210 DAP (Figure 2 and Supplementary Table S3). Plants inoculated with SSL C3.11 and C3.14 had a higher PI than their parent C3 or their sibling C3.12 (Figure 2 and Supplementary Table S3). This result shows that plant physiological responses also vary among plants inoculated with clonally produced siblings of a dikaryon mycorrhizal fungus. None of the treatments differed from either of the two controls Ca and No. F_v_/F_m_ and PI were closely correlated (R^2^ = 0.7279, *p* < 0.0001). AMF effects on Chl *a* fluorescence at 210 DAP were not correlated with either root productivity or AMF colonization at the final harvest (data not shown).

**TABLE 1:** Means (± standard error) of PSII (Fv/Fm), performance index (PI), leaf water potential (LWP) and soil moisture (SM) of cassava at different times (120, 210 and 300 DAP) in Experiment 1 and the results of a repeated measures analysis on these variables to test for a sampling time effect.

**Sampling time**		**Fv/Fm**	**PI**	**LWP (MPa)**	**SM (% v/v)**
120 DAP		0.80 ± 0.00 a	1.61 ± 0.09 a	−0.49 ± 0.01 b	25.21 ± 0.22 a
210 DAP		0.75 ± 0.00 b	0.85 ± 0.04 c	−0.52 ± 0.01 b	13.55 ± 0.19 b
300 DAP		0.80 ± 0.00 a	1.38 ± 0.05 b	−0.37 ± 0.01 a	24.20 ± 0.48 a

	**df**	***F* value**			

Inoculation	14	0.7667 ns	0.8561 ns	0.9074 ns	0.6923 ns
Sampling time	2	36.5801**	41.1201**	52.5103**	11.1431**
Inoculation x sampling time	28	0.9989 ns	1.3540 ns	0.4953 ns	0.4771 ns
Mean		0.79	1.28	−0.46	20.65
CV (%)		5	58.25	−29.91	20.51

The table shows the F ratios of each of the factors. Means with different letters are significantly different according to Tukey post-hoc test (*p* < 0.05). * = *p* < 0.05; ** = *p* < 0.01; ns = no significant; CV (%) = variation coefficient.

### Experiment 2. Effects of *R. irregularis* Isolates on Cassava Drought Stress Responses and Recovery

#### Effects of Drought on Cassava Growth

**FIGURE 3:**
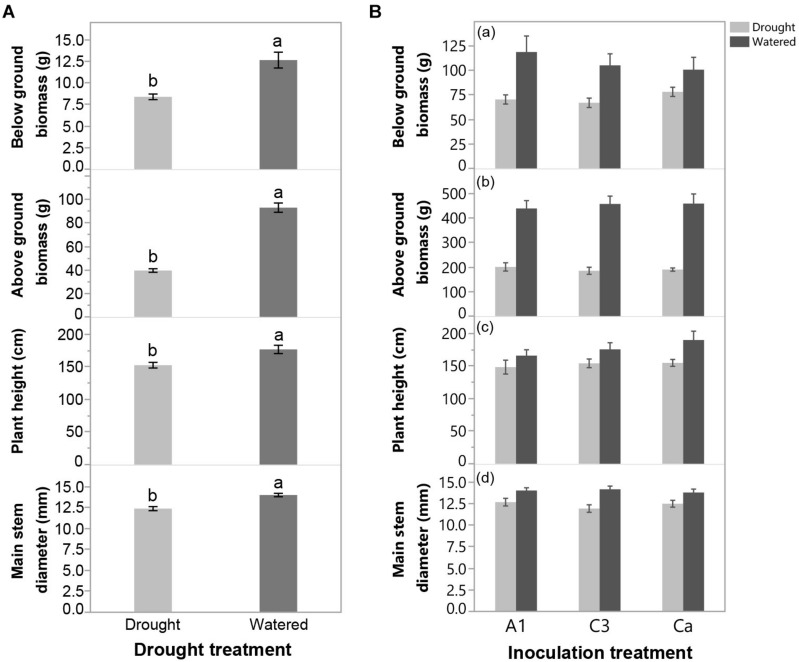
**(A)** Effect of drought treatment on below ground biomass, above ground biomass, plant height and main stem diameter at the end of Experiment 2 (greenhouse trial). **(B)** Effect of inoculation with *R. irregularis* isolates C3 and A1 on below ground biomass, above ground biomass, plant height and main stem diameter at the end of Experiment 2 in either Watered or Drought treatments in Experiment 2 (greenhouse trial). Error bars represent ± S.E. Different letters above bars represent significant differences at *p* < 0.05 according to a Tukey post-hoc test.

Withholding water at 110 DAP resulted in significant detrimental effects on above-ground biomass, below ground biomass, plant height and main stem diameter of cassava (Figure 3a and Supplementary Table S4), indicating that plants experienced significant drought stress. There were no significant differences in any of the cassava growth variables among inoculation treatments and there was no significant inoculation x drought interaction (Figure 3b and Supplementary Table S4).

#### Effects of Drought and AMF Isolates on Gas Exchange and Chlorophyll *a* Fluorescence

The variables photosynthetic capacity (A_max_), stomatal conductance (g_smax_), water use efficiency (WUE), maximum quantum yield of PSII (F_v_/F_m_) and performance index (PI) significantly differed between drought treatments at 116 DAP (6 days after withholding of water). These differences were sustained until the harvest of the experiment (Table 2). The largest differences in gas exchange and Chl *a* fluorescence between drought-treated and well-watered plants were observed at 119 DAP (point of maximum withholding of water) and 123 DAP (3 days after re-watering), respectively (Figure 1 and Table 2).

**TABLE 2:** Means (± standard error) of photosynthetic capacity (A_max_), stomatal conductance (gs_max_), water use efficiency (WUE), maximum quantum yield of PSII (Fv/Fm) and performance index (PI) of cassava to test for a drought effect between plants in well-watered (watered) and water-stressed (drought) conditions at each sampling time in Experiment 2 (greenhouse trial).

**Sampling time (DAP)**	**Soil moisture (% v/v)**		**A_max_ (μmol m^–2^ s^–1^)**		**gs_max_ (mmol m^–2^ s^–1^)**		**WUE**		**Fv/Fm**		**PI**	
						
	**Watered**	**Drought**	**Watered**	**Drought**	**Watered**	**Drought**	**Watered**	**Drought**	**Watered**	**Drought**	**Watered**	**Drought**
103	25.00	25.00	5.730.67	5.720.66	23.333.32	18.672.26	0.290.03	0.310.02	0.820.01	0.820.01	2.840.24	2.840.27
109	23.18	23.18	7.750.75	7.970.72	56.876.80	63.935.28	0.150.02	0.130.01	0.810.01	0.820.01	2.820.30	2.780.30
116	31.30	10.66	7.550.51	3.360.89**	47.804.29	32.278.46	0.160.01	0.10.03*	0.790.01	0.810.01	1.650.30	2.060.23
119	28.56	6.66	7.140.40	−0.450.22**	38.513.98	6.21.56**	0.280.09	0.000.00**	0.840.00	0.770.01**	3.440.18	1.760.32**
123	32.60	9.80	7.130.40	−0.170.14**	39.23.77	6.331.70**	0.200.02	−0.020.01**	0.810.01	0.750.01**	2.330.13	1.120.15**
130	31.14	12.00	4.090.32	2.580.38**	15.801.93	9.131.38**	0.300.03	0.320.06	0.800.03	0.780.01*	1.740.15	1.210.12*
136	38.21	26.00	4.390.29	3.370.30*	33.672.7	21.271.35**	0.140.01	0.160.01	0.810.01	0.790.01*	2.420.13	1.670.14**
140	32.70	35.85	6.020.41	7.60.56*	35.272.35	39.473.62	0.180.01	0.210.02	0.820.00	0.800.01*	2.240.11	1.510.13**

Estimations followed by *, **, were significant at the level of *p* < 0.05 and *p* < 0.01, respectively, compared to the well-watered plants (watered), according to a Dunnett’s test.

**FIGURE 4:**
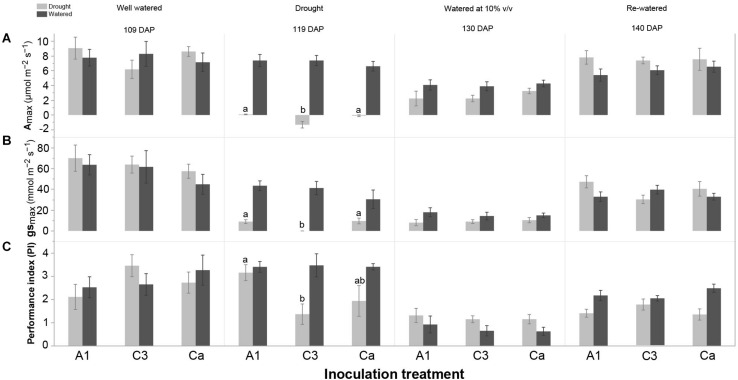
Effect of inoculation with *R. irregularis* isolates A1 and C3 on **(A)** photosynthetic capacity (A_max_), **(B)** stomatal conductance (g_smax_) and **(C)** performance index (PI) of cassava in well-watered (watered) and water-stressed (drought) conditions at four sampling times: 109 DAP, 119 DAP, 130 DAP and 140 DAP in Experiment 2. Error bars represent ± S.E. Different letters above bars represent significant differences at *p* < 0.05 according to a Tukey post-hoc test. Comparison among inoculation treatments in either well-watered (watered) or water-stressed (drought) conditions at each sampling time.

We further analyzed the effect of the inoculation treatments on gas exchange and Chl *a* fluorescence of cassava in watered and drought conditions at each sampling time (Figure 4 and Supplementary Table S5). At 119 DAP (point of maximum withholding of water), in plants subjected to drought, C3 inoculated plants had a lower A_max_, g_s__max_ and PI than plants inoculated with A1 and the control Ca (Figure 4 and Supplementary Table S5). WUE and F_v_/F_m_ did not differ among the inoculation treatments at 119 DAP in cassava subjected to drought (Supplementary Table S5). There was no clear effect of AMF inoculation on gas exchange and Chl *a* fluorescence measurements at other sampling times in plants subjected to drought. There was also no inoculation treatment effect on gas exchange and Chl *a* fluorescence measurements at any of sampling times in well-watered plants (Supplementary Table S5).

#### AMF Isolate Effects on Recovery Rate

**FIGURE 5:**
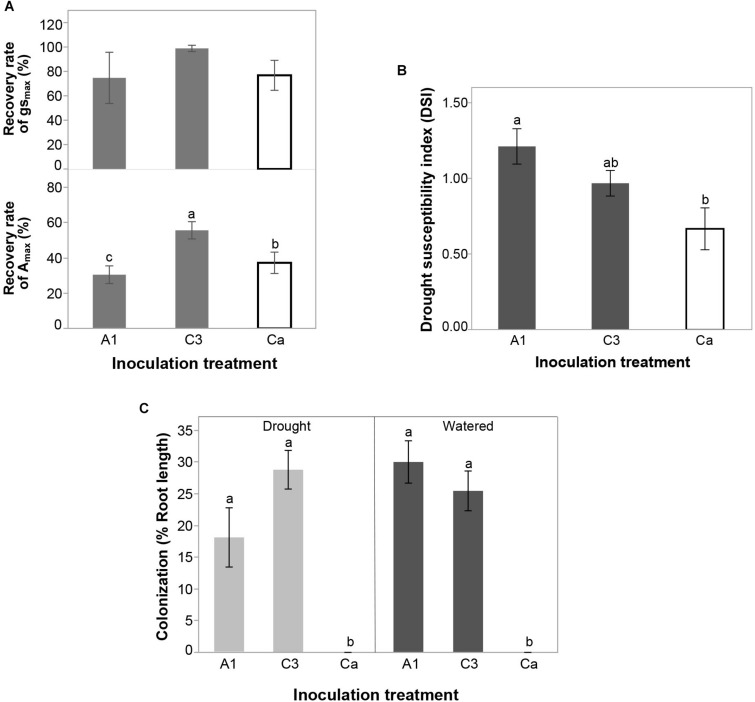
**(A)** Effect of inoculation with *R. irregularis* isolates A1 and C3 on the recovery rate of stomatal conductance (g_smax_) and photosynthetic capacity (A_max_) during re-watering period in plants that had been subjected to drought in Experiment 2. **(B)** Effect of inoculation with *R. irregularis* isolates A1 and C3 on the drought susceptibility index (DSI) of cassava in Experiment 2. **(C)** Effect of inoculation with *R. irregularis* isolates A1 and C3 on AMF colonization (% root length) in water-stressed (drought) and well-watered (watered) conditions in Experiment 2. Error bars represent ± S.E. Different letters above bars represent significant differences at *p* < 0.05 according to a Tukey post-hoc test.

The recovery rate of A_max_ was higher in plants inoculated with C3 compared to plants inoculated with A1 and the control Ca (Figure 5a). The recovery rate of g_smax_ did not differ among inoculation treatments, although the trend was the same as that observed for photosynthetic capacity. There were also no inoculation effects on the recovery rate of any of the variables measured in the Chl *a* fluorescence (Supplementary Figure S2).

#### AMF Isolate Effects on Drought Tolerance

At the end of the experiment, plants inoculated with A1 were significantly more susceptible to drought than control Ca (Figure 5b and Supplementary Table S6). C3 treatment did not differ from the other two inoculation treatments. There was no significant inoculation treatment effect on RYR (Supplementary Table S6).

#### AMF Colonization in Drought-Treated and Control Plants

Plants inoculated with *R. irregularis* isolates A1 and C3 were equally colonized in both well-watered and drought conditions and there was no difference in colonization between drought treatments (Figure 5c).

## Discussion

We showed that in field conditions, cassava inoculated with genetically different parental *R. irregularis* isolates or their progeny displayed significantly different stress levels. In the second experiment, plants experienced drought as an experimental treatment allowing us to better understand the underlying physiological processes that were observed in field conditions. We observed in controlled conditions that the photosynthetic responses of cassava during drought and recovery depended heavily on which AMF isolate colonized the cassava roots. The physiological response to drought was mainly controlled by stomatal processes before non-stomatal limitation at the cellular level could permanently damage the photosynthetic apparatus.

### Cassava Inoculated With *R. irregularis* Parental Isolates and Their Clonal Progeny Respond Differently to Stress

In the first experiment, plants experienced a natural dry period between 120 and 300 DAP. We assessed plant stress at these times by measuring the Chl *a* fluorescence variables F_v_/F_m_ and PI because these are variables commonly measured in such experiments. F_v_/F_m_ represents the efficiency of energy flow at PSII, commonly used as an indicator of plant photosynthetic efficiency under stress conditions (Maxwell and Johnson, 2000). PI represents the efficiency in the energy flux through the different phases of electron transport inside the photosynthetic apparatus, providing quantitative information on the efficiency of PSII and PSI that is normally reduced during stress (Strasser et al., 2000, 2004; Silvestre et al., 2014). Low F_v_/F_m_ and PI values indicate that plants are stressed.

Chlorophyll *a* fluorescence measurements observed in the dry period (at 210 DAP) indicated that the plants experienced stress. Similar stress responses were induced experimentally in the 2^nd^ experiment. F_v_/F_m_ values below this threshold indicate a reduction in PSII efficiency and, consequently, plant stress (Maxwell and Johnson, 2000 and Oyetunji et al., 2007). Mean F_v_/F_m_ measurements indicated that cassava experienced stress in the field throughout the experiment, but were most stressed at 210 DAP, coinciding with natural drought. PI values at 210 DAP were consistent with those reported in other plant species under severe drought (Silvestre et al., 2014). These values of Chl *a* fluorescence at 210 DAP could mainly be attributed to low accumulated precipitation in the previous 30 days before sampling.

Variation in plant responses to stress due to inoculation with different individuals of one AMF species has not previously been reported although interspecific AMF effects have been observed (Oyetunji et al., 2007; Rai et al., 2008; Chitarra et al., 2016). Inoculating cassava with different *R. irregularis* cultures altered the stress experienced by cassava in farming conditions because F_v_/F_m_ and PI values differed significantly and strongly among inoculation treatments. These values were even lower than those observed in plants subjected experimentally to severe drought during the second experiment. PI is more sensitive to changes in stress than Fv/Fm (Oukarroum et al., 2007; Živčák et al., 2008). Indeed, inoculation with either C2 or C3, or their progeny, greatly affected PI by up to a 2.58-fold difference, respectively. PI at 210 DAP of individual treatments was even lower than values reported in the second experiment where plants were subjected to severe drought. Progeny of parents C2 and C3 were also shown to significantly affect cassava growth (data reported in Ceballos et al., 2019). AMF colonization of the roots at the final harvest differed among C3 and its progeny but not among C2 and its progeny (Ceballos et al., 2019). Neither the AMF effects on root productivity nor AMF colonisation at the final harvest were correlated with Chl *a* fluorescence at 210 DAP.

The results show that clonal progeny of parental isolates C2 and C3 made cassava respond differently to stress. However, we cannot confirm that these differences are due to genetic differences among progeny because offspring of isolate C2 (a homokaryon) should be genetically identical.

### Genetically Different *R. irregularis* Parental Isolates Induce Differential Cassava Physiological Responses to Drought Stress and Recovery From Drought

In the first experiment, we observed that stress in cassava is influenced by AMF identity and that stress was likely a response to drought. In the second experiment plants were experimentally subjected to drought allowing us to demonstrate that indeed genetically different isolates induce differential responses of cassava to drought.

Withholding water at 110 DAP had the expected effect affecting most of the physiological measurements from 116 DAP until the end of the experiment. Inoculation with isolates A1 and C3 largely altered the response of cassava during drought; mostly because C3 inoculated plants suffered more than plants inoculated with A1 and the control, and even showing negative photosynthetic rates. However, drought affected plants inoculated with C3 exhibited a higher recovery rate during re-watering, compared to A1 and control plants. Drought did not cause permanent damage on stomatal control, as it mostly only induced stomatal limitation of photosynthesis. Interestingly, that limitation was quickly reversed in all drought treated plants, but faster in C3 inoculated plants. Non-stomatal limitation of photosynthesis, would induce permanent damage to the photosystem that would also result in a slower recovery rate. Surprisingly, although inoculation did not directly affect plant biomass at harvest, drought affected plants inoculated with A1 were more susceptible to drought than the control (Ca), as measured by the DSI. The lack of an effect on biomass may be due to the length of the experiment (5 months) which many not have been sufficiently long to detect biomass differences. The interpretation of DSI should be taken with some caution because plant genotypes with higher drought tolerance are not always the most productive (Cardona-Ayala et al., 2014).

From the result of this study, we cannot say why genetically different *R. irregularis* isolates alter cassava stress responses to drought. Differences exist between AMF isolates C3 and A1 in extra-radicle hyphal length and hyphal growth rates Koch et al. (2004). It is known that extra-radicle AMF hyphal length is positively correlated with soil aggregation and that soil aggregation controls the water movement (Rillig et al., 2010, 2014). Consequently, it is possible that intraspecific variation in fungal traits like extraradical hyphal length could have an indirect effect on soil water availability, which in turn could affect plant susceptibility to drought.

These results indicate that intraspecific genetic variation, and possibly epigenetic variation, in *R. irregularis* influence physiological responses of cassava to water stress. These results were observed independently in the field and in the greenhouse. To our knowledge, that variation in an AMF species influences cassava drought responses differently has not previously been reported. This research does not specifically highlight any AMF isolate as having an outright negative or positive effect on cassava because response to inoculation is dependent on multiple factors, including cassava variety and local environmental conditions (Ceballos et al., 2019). However, results indicate that inoculation with different AMF of the same species is likely to alter cassava responses to drought in the field, even though plants naturally become colonized by the local mycorrhizal fungal community as well. A better understanding of how variation in *R. irregularis* affects responses to stress during drought could offer further possibilities to increase yield and stress tolerance in cassava in areas where cassava experiences strong drought conditions during part of the cropping period. The results confirm that within-fungus genetic variability in *R. irregularis* should be considered. AMF variation could be relevant for future applications in agriculture, particularly in areas affected by harsh environmental conditions or impending climate change. Better understanding of the different molecular layers of *R. irregularis*, from genome organization to gene transcription, and how they contribute to host health are necessary to select the most beneficial candidate AMF lines.

## Data Availability Statement

The raw data supporting the conclusions of this article will be made available by the authors, without undue reservation.

## Author Contributions

RP conceived and conducted Experiments 1 and 2, analyzed and interpreted the data, and wrote the manuscript. CR conceived and conducted Experiment 2, interpreted the data, and wrote the manuscript. JC conceived and conducted Experiment 2. MT conceived and conducted Experiment 1. CM and BV conceived Experiment 1. CS conducted a part of Experiment 2, interpreted the data, and wrote the manuscript. AR and IS conceived Experiments 1 and 2, interpreted the data, and wrote the manuscript. All authors contributed to the article and approved the submitted version.

## Conflict of Interest

The authors declare that the research was conducted in the absence of any commercial or financial relationships that could be construed as a potential conflict of interest.

## References

[B1] Aliyu I. A. Yosef A. A. Uyovbisere E. O. Masso C. Sanders I. R. (2019). Effect of co-application of phosphorus fertilizer and in vitro-produced mycorrhizal fungal inoculants on yield and leaf nutrient concentration of cassava. *PLoS One* 14:e0218969. 10.1371/journal.pone.0218969 31242274 PMC6594633

[B2] Angelard C. Colard A. Niculita-Hirzel H. Croll D. Sanders I. R. (2010). Segregation in a mycorrhizal fungus alters rice growth and symbiosis-specific gene transcription. *Curr. Biol.* 20 1216–1221. 10.1016/j.cub.2010.05.031 20541408

[B3] Augé R. M. (2001). Water relations, drought and vesicular-arbuscular mycorrhizal symbiosis. *Mycorrhiza* 11 3–42. 10.1007/s005720100097

[B4] Boldt K. Pörs Y. Haupt B. Bitterlich M. Kühn C. Grimm B. (2011). Photochemical processes, carbon assimilation and RNA accumulation of sucrose transporter genes in tomato arbuscular mycorrhiza. *J. Plant Physiol.* 168 1256–1263. 10.1016/j.jplph.2011.01.026 21489650

[B5] Cardona-Ayala C. Jarma-Orozco A. Araméndiz-Tatis H. Peña-Agresott M. Vergara-Córdoba C. (2014). Physiological and biochemical responses of the cowpea bean (*Vigna unguiculata* L. Walp.) under a water deficit. *Rev. Colomb. Cienc. Hortic.* 8 250–261.

[B6] Ceballos I. Mateus I. D. Peña R. Peña-Quemba D. C. Robbins C. Ordoñez Y. M. (2019). Using variation in arbuscular mycorrhizal fungi to drive the productivity of the food security crop cassava. *bioRxiv* [Preprint]. 10.1101/830547

[B7] Ceballos I. Ruiz M. Fernández C. Peña R. Rodríguez A. Sanders I. R. (2013). The in vitro mass-produced model mycorrhizal fungus, rhizophagus irregularis, significantly increases yields of the globally important food security crop cassava. *PLoS One* 8:e70633. 10.1371/journal.pone.0070633 23950975 PMC3737348

[B8] Chen E. C. H. Morin E. Deaudet D. Noel J. Yildirir G. Ndikumana S. (2018). High intraspecific genome diversity in the model arbuscular mycorrhizal symbiont Rhizophagus irregularis. *New Phyt.* 220 1161–1171. 10.1111/nph.14989 29355972

[B9] Chitarra W. Pagliarani C. Maserti B. Lumini E. Siciliano I. Cascone P. (2016). Insights on the impact of arbuscular mycorrhizal symbiosis on tomato tolerance to water stress. *Plant Physiol.* 171 1009–1023. 10.1104/pp.16.00307 27208301 PMC4902612

[B10] Czyczyło-Mysza I. Tyrka M. Marcińska I. Skrzypek E. Karbarz M. Dziurka M. (2013). Quantitative trait loci for leaf chlorophyll fluorescence parameters, chlorophyll and carotenoid contents in relation to biomass and yield in bread wheat and their chromosome deletion bin assignments. *Mol Breed.* 32 189–210. 10.1007/s11032-013-9862-8 23794940 PMC3684715

[B11] De Oliveira E. J. Morgante C. V. De Tarso Aidar S. de Melo Chaves A. R. Antonio R. P. Cruz J. L. (2017). Evaluation of cassava germplasm for drought tolerance under field conditions. *Euphytica* 213:188 10.1007/s10681-017-1972-7

[B12] De Ronde J. A. Cress W. A. Krüger G. H. J. Strasser R. J. Van Staden J. (2004). Photosynthetic response of transgenic soybean plants, containing an Arabidopsis P5CR gene, during heat and drought stress. *J. Plant Physiol.* 161 1211–1224. 10.1016/j.jplph.2004.01.014 15602813

[B13] Ehinger M. Croll D. Koch M. A. Sanders I. R. (2012). Significant genetic and phenotypic changes arising from clonal growth of a single spore of an arbuscular mycorrhizal fungus over multiple generations. *New Phytol.* 196 853–861. 10.1111/j.1469-8137.2012.04278.x 22931497

[B14] Ekanayake I. J. Oyetunji O. J. Osonubi O. Lyasse O. (2004). The effects of arbuscular mycorrhizal fungi and water stress on leaf chlorophyll production of cassava (*Manihot esculenta* Crantz). *J. Food, Agric. Environ.* 2 190–196.

[B15] El-Sharkawy M. A. (2006). International research on cassava photosynthesis, productivity, eco-physiology, and responses to environmental stresses in the tropics. *Photosynthetica* 44 481–512. 10.1007/s11099-007-0067-4

[B16] El-Sharkawy M. A. (2014). Global warming: causes and impacts on agroecosystems productivity and food security with emphasis on cassava comparative advantage in the tropics/subtropics. *Photosynthetica* 52:161 10.1007/s11099-014-0028-7

[B17] FAO (2012). *Coping with Water Scarcity an Action Framework for Agriculture and Food Security.* Rome: FAO.

[B18] FAO (2013). *Save and Grow: Cassava. A Guide to Sustainable Production Intensification.* Rome: FAO.

[B19] Flexas J. Bota J. Loreto F. Cornic G. Sharkey T. D. (2004). Diffusive and metabolic limitations to photosynthesis under drought and salinity in C-3 plants. *Plant Biol.* 6 269–279. 10.1055/s-2004-820867 15143435

[B20] García-Sánchez M. Palma J. M. Ocampo J. A. García-Romera I. Aranda E. (2014). Arbuscular mycorrhizal fungi alleviate oxidative stress induced by ADOR and enhance antioxidant responses of tomato plants. *J. Plant Physiol.* 171 421–428. 10.1016/j.jplph.2013.10.023 24594394

[B21] Giovanetti M. Mosse B. (1980). An evaluation of techniques for measuring vesicular mycorrhizal infection in roots. *New Phytol.* 97 447–453.

[B22] Grassi G. Magnani F. (2005). Stomatal, mesophyll conductance and biochemical limitations to photosynthesis as affected by drought and leaf ontogeny in ash and oak trees. *Plant Cell Environ.* 28 834–849. 10.1111/j.1365-3040.2005.01333.x

[B23] Grzesiak M. T. Waligórski P. Janowiak F. Marcińska I. Katarzyna H. (2013). The relations between drought susceptibility index based on grain yield (DSIGY) and key physiological seedling traits in maize and triticale genotypes. *Acta Physiol. Plant* 35:549 10.1007/s11738-012-1097-5

[B24] Harrison M. J. (1999). Molecular and cellular aspects of the arbuscular mycorrhizal symbiosis. *Annu. Rev. Plant Physiol. Plant Mol. Biol.* 50 361–389. 10.1146/annurev.arplant.50.1.361 15012214

[B25] Hu W. Zhang H. Chen H. Tang M. (2017). Arbuscular mycorrhizas influence Lycium barbarum tolerance of water stress in a hot environment. *Mycorrhiza* 27 451–463. 10.1007/s00572-017-0765-0 28185001

[B26] IPCC (2018). *Global Warming of 1.5 °C: An IPCC Special Report on The Impacts of Global Warming of 1.5 °C Above Pre-Industrial Levels and Related Global Greenhouse Gas Emission Pathways, in The Context of Strengthening the Global Response to The Threat of Climate Change, Sustainable Development, and Efforts to Eradicate Poverty.* Geneva: Intergovernmental Panel on Climate Change.

[B27] Kalaji H. M. Jajoo A. Oukarroum A. Brestic M. Zivcak M. Samborska I. A. (2016). Chlorophyll a fluorescence as a tool to monitor physiological status of plants under abiotic stress conditions. *Acta Physiol. Plant.* 38:102.

[B28] Keymer A. Pimprikar P. Wewer V. Huber C. Brands M. Bucerius S. L. (2017). Lipid transfer from plants to arbuscular mycorrhiza fungi. *eLife* 6:e29107. 10.7554/eLife.29107 28726631 PMC5559270

[B29] Koch A. M. Antunes P. M. Maherali H. Hart M. M. Klironomos J. N. (2017). Evolutionary asymmetry in the arbuscular mycorrhizal symbiosis : conservatism in fungal morphology does not predict host plant growth. *New Phyt.* 214 1330–1337. 10.1111/nph.14465 28186629

[B30] Koch A. M. Croll D. Sanders I. R. (2006). Genetic variability in a population of arbuscular mycorrhizal fungi causes variation in plant growth. *Ecol. Lett.* 9 103–110. 10.1111/j.1461-0248.2005.00853.x 16958874

[B31] Koch A. M. Kuhn G. Fontanillas P. Fumagalli L. Goudet J. Sanders I. R. (2004). High genetic variability and low local diversity in a population of arbuscular mycorrhizal fungi. *PNAS* 101 2369–2374. 10.1073/pnas.0306441101 14983016 PMC356957

[B32] Koske R. E. Gemma J. N. (1989). A modified procedure for staining roots to detect VA mycorrhizas. *Mycol. Res.* 92 486–488. 10.1016/s0953-7562(89)80195-9

[B33] Masclaux F. G. Wyss T. Mateus I. D. Aletti C. Sanders I. R. (2018). Variation in allele frequencies at the bg112 locus reveals unequal inheritance of nuclei in a dikaryotic isolate of the fungus *Rhizophagus irregularis*. *Mycorrhiza* 28 369–377. 10.1007/s00572-018-0834-z 29675619

[B34] Masclaux F. G. Wyss T. Pagni M. Rosikiewicz P. Sanders I. R. (2019). Investigating unexplained genetic variation and its expression in the arbuscular mycorrhizal fungus *Rhizophagus irregularis*: a comparison of whole genome and RAD sequencing data. *PLoS One* 14:e0226497. 10.1371/journal.pone.0226497 31881076 PMC6934306

[B35] Maxwell K. Johnson G. N. (2000). Chlorophyll fluorescence—a practical guide. *J. Exp. Bot.* 51 659–668. 10.1093/jxb/51.345.659 10938857

[B36] Müller P. Xiao-Ping L. Niyogi K. K. (2001). Non-photochemical quenching. A response to excess light energy. *Plant Physiol.* 125 1558–1566. 10.1104/pp.125.4.1558 11299337 PMC1539381

[B37] Munkvold L. Kjoller R. Vestberg M. Rosendahl S. Jakobsen I. (2004). High functional diversity within species of arbuscular mycorrhizal fungi. *New Phytol.* 164 357–364. 10.1111/j.1469-8137.2004.01169.x 33873553

[B38] Niu G. Rodriguez D. S. (2008). Growth and physiological responses to drought stress in four oleander clones. *J. Am. Soc. Hort. Sci.* 133 188–196. 10.21273/jashs.133.2.188

[B39] Okogbenin E. Setter T. L. Ferguson M. Mutegi R. Ceballos H. Olasanmi B. (2013). Phenotypic approaches to drought in cassava: review. *Front. Physiol.* 4:93. 10.3389/fphys.2013.00093 23717282 PMC3650755

[B40] Oukarroum A. Madidi S. E. Schansker G. Strasser R. J. (2007). Probing the responses of barley cultivars (*Hordeum vulgare* L.) by chlorophyll a fluorescence OLKJIP under drought stress and re-watering. *Environ. Exp. Bot.* 60 438–446. 10.1016/j.envexpbot.2007.01.002

[B41] Oyetunji O. J. Ekanayake I. J. Osonubi O. (2007). Chlorophyll fluorescence analysis for assessing water deficit and arbuscular mycorrhizal fungi (AMF) inoculation in cassava (*Manihot esculenta* Crantz). *Adv. Biol. Res.* 1 108–117.

[B42] Peirone L. S. Pereyra Irujo G. A. Bolton A. Erreguerena I. Aguirrezábal L. A. N. (2018). Assessing the Efficiency of Phenotyping Early Traits in a Greenhouse Automated Platform for Predicting Drought Tolerance of Soybean in the Field. *Front. Plant Sci.* 9:587. 10.3389/fpls.2018.00587 29774042 PMC5943574

[B43] Rai M. K. Shende S. Strasser R. J. (2008). JIP test for fast fluorescence ransients as a rapid and sensitive technique in assessing the effectiveness of arbuscular mycorrhizal fungi in *Zea mays*: analysis of chlorophyll a fluorescence. *Plant Biosyst. Int. J. Dealing All Aspects Plant Biol.* 142 191–198. 10.1080/11263500802150225

[B44] Rillig M. C. Aguilar-Trigueros C. A. Bergmann J. Verbruggen E. Veresoglou S. D. Lehmann A. (2014). Plant root and mycorrhizal fungal traits for understanding soil aggregation. *New Phytol.* 205 1385–1388. 10.1111/nph.13045 25231111

[B45] Rillig M. C. Mardatin N. F. Leifheit E. F. Antunes P. M. (2010). Mycelium of arbuscular mycorrhizal fungi increases soil water repellency and is sufficient to maintain water-stable soil aggregates. *Soil Biol. Biochem.* 42 1189–1191. 10.1016/j.soilbio.2010.03.027

[B46] Rodriguez A. Sanders I. R. (2015). The role of community and population ecology in applying mycorrhizal fungi for improved food security. *ISME J.* 9 1053–1061. 10.1038/ismej.2014.207 25350159 PMC4409159

[B47] Savary R. Masclaux F. G. Droh G. Cruz-Corella J. Machado A. P. Morton J. B. (2018). A population genomics approach shows widespread geographical distribution of cryptic genomic forms of the symbiotic fungus *Rhizophagus irregularis*. *ISME J.* 12 17–30. 10.1038/ismej.2017.153 29027999 PMC5739010

[B48] Scholander P. F. Hammel H. T. Hemmingsen E. A. Bradstreet E. D. (1964). Hydrostatic pressure and osmotic potential in leaves of mangroves and some other plants. *Proc. Natl. Acad. Sci. U.S.A.* 52 119–125. 10.1073/pnas.52.1.119 16591185 PMC300583

[B49] Séry D. J. M. Kouadjo Z. G. C. Voko B. R. R. Zézé A. (2016). Selecting native arbuscular mycorrhizal fungi to promote cassava growth and increase yield under field conditions. *Front. Microbiol.* 7:2063. 10.3389/fmicb.2016.02063 28066381 PMC5177653

[B50] Signarbieux C. Feller U. (2011). Non-stomatal limitations of photosynthesis in grassland species under artificial drought in the field. *Environ. Exp. Bot.* 71 192–197. 10.1016/j.envexpbot.2010.12.003

[B51] Silvestre S. de Sousa Araújo S. Vaz Patto M. C. Marques da Silva J. (2014). Performance index: an expeditious tool to screen for improved drought resistance in the *Lathyrus* genus. *J. Integr. Plant Biol.* 56 610–621. 10.1111/jipb.12186 25126659

[B52] St-Arnaud M. Vujanovic V. (2006). “Effect of the arbuscular mycorrhizal symbiosis on plant diseases and pests,” in *Mycorrhizae in Crop Production: Applying Knowledge*, eds Hamel C. Plenchette C. (Binghamton, NY: Haworth Press).

[B53] Strasser R. J. Srivastava A. Tsimilli-Michael M. (2000). “The fluorescence transient as a tool to characterize and screen photosynthetic samples,” in *Probing Photosynthesis: Mechanisms, Regulation and Adaptation*, eds Yunus M. Pathre U. Mohanty P. (London: Taylor & Francis Publishers), 445–483.

[B54] Strasser R. J. Tsimilli-Michael M. Srivastava A. (2004). “Analysis of the chlorophyll a fluorescence transient,” in *Chlorophyll a Fluorescence: A Signature of Photosynthesis*, eds Papadogeorgiou G. Govindjee C. (The Netherlands: Springer), 321–362. 10.1007/978-1-4020-3218-9_12

[B55] Szilas C. Koch C. B. Msolla M. M. Borggaard O. K. (2008). The reactivity of Tanzanian Minjingu phosphate rock can be assessed from the chemical and mineralogical composition. *Geoderma* 147 172–177. 10.1016/j.geoderma.2008.08.009

[B56] Tsimilli-Michael M. Strasser R. J. (2008). “In vivo assessment of plants’ vitality: applications in detecting and evaluating the impact of mycorrhization on host plants,” in *Mycorrhiza: Genetics and Molecular Biology, Eco-Function, Biotechnology, Eco-Physiology, Structure and Systematics*, 3rd Edn, ed. Varma A. (Cham: Springer), 679–703. 10.1007/978-3-540-78826-3_32

[B57] Wang W. Vinocur B. Altman A. (2003). Plant responses to drought, salinity and extreme temperatures: towards genetic engineering for stress tolerance. *Planta* 218 1–14. 10.1007/s00425-003-1105-5 14513379

[B58] Wyss T. Masclaux F. G. Rosikiewicz P. Pagni M. Sanders I. R. (2016). Population genomics reveals that within-fungus polymorphism is common and maintained in populations of the mycorrhizal fungus Rhizophagus irregularis. *ISME J.* 10 2514–2526. 10.1038/ismej.2016.29 26953600 PMC5030683

[B59] Živčák M. Brestič M. Olšovská K. Slamka P. (2008). Performance index as a sensitive indicator of water stress in *Triticum aestivum* L. *Plant Soil Environ.* 54 133–139. 10.17221/392-PSE

[B60] Zushi K. Matsuzoe N. (2017). Using of chlorophyll a fluorescence OJIP transients for sensing salt stress in the leaves and fruits of tomato. *Sci. Hortic.* 219 216–221. 10.1016/j.scienta.2017.03.016

