# Supplementary material for: Genetically Different Isolates of the Arbuscular Mycorrhizal Fungus Rhizophagus irregularis Induce Differential Responses to Stress in Cassava
Source: Front Plant Sci. 2020 Dec 2;11:596929. doi: 10.3389/fpls.2020.596929 (PMC7793890; doi:10.3389/fpls.2020.596929)
Supplement: Supplementary file 1 [file Data_Sheet_1.DOCX]

**Genetically Different Isolates of the Arbuscular Mycorrhizal Fungus *Rhizophagus irregularis* Induce Differential Responses to Stress in Cassava (Peña *et al*.)**

**SUPPLEMENTARY INFORMATION**

This document contains supplementary tables 1-6 and supplementary figures 1-2.

**Table S1.** Names of single spore line (SSL) progeny of parental R. *irregularis* isolates (C2 and C3) used in Experiment 1 (field trial). Codenames of single spore lines start with the name of the parental isolate and the number following the point denotes the single spore used to initiate the culture. We included R. *irregularis* isolate C5 which has been shown to be a clone of C2 (Wyss *et al.,* 2016). Cassava was inoculated with the parental isolate, plus its single spore progeny.

| **Parental lines** | **C2/C5** | **C3** |
| --- | --- | --- |
|  | Monokaryon | Dikaryon |
| **Single spore lines (SSLs)** | C2.5 | C3.10 |
|  | C2.6 | C3.11 |
|  | C2.7 | C3.12 |
|  | C2.8 | C3.14 |
|  |  | C3.15 |
|  |  | C3.16 |

**Table S2.** Results of one-way ANOVA on maximum quantum yield of PSII (Fv/Fm), performance index (PI), leaf water potential (LWP) and soil moisture (SM) to test for an inoculation treatment effect on cassava inoculated with parental isolate C2 and its offspring at each sampling time (120, 210 and 300 DAP) in Experiment 1 (field trial). Means (± standard error) of highlighted tests are plotted in Figure 2.

| **Sampling time** | **df** | **SS** | **F ratio** | ***p*** |
| --- | --- | --- | --- | --- |
|  | **One-way ANOVA on Fv/Fm** | | | |
| 120 DAP | 7 | 0.0030 | 0.4111 | 0.8911 |
| 210 DAP | 7 | 0.0364 | 10.3775 | 0.0001 |
| 300 DAP | 7 | 0.0037 | 0.9622 | 0.4675 |
|  |  |  |  |  |
|  | **One-way ANOVA on PI** | | | |
| 120 DAP | 7 | 3.7561 | 0.6339 | 0.7256 |
| 210 DAP | 7 | 4.8555 | 7.5765 | 0.0001 |
| 300 DAP | 7 | 2.9511 | 1.1057 | 0.3724 |
|  |  |  |  |  |
|  | **One-way ANOVA on LWP (MPa)** | | | |
| 120 DAP | 7 | 0.1257 | 1.3572 | 0.2424 |
| 210 DAP | 7 | 0.0551 | 0.3392 | 0.9324 |
| 300 DAP | 7 | 0.0677 | 1.6696 | 0.1354 |
|  |  |  |  |  |
|  | **One-way ANOVA on SM (% v/v)** | | | |
| 120 DAP | 7 | 14.5937 | 0.3177 | 0.9428 |
| 210 DAP | 7 | 24.4402 | 0.7213 | 0.6544 |
| 300 DAP | 7 | 27.5947 | 0.2666 | 0.9643 |
|  |  |  |  |  |

**Table S3.** Results of one-way ANOVA on maximum quantum yield of PSII (Fv/Fm), performance index (PI), leaf water potential (LWP) and soil moisture (SM) to test for an inoculation treatment effect on cassava inoculated with parental isolate C3 and its offspring at each sampling time (120, 210 and 300 DAP) in Experiment 1 (field trial). Means (± standard error) of highlighted tests are plotted in Figure 2.

| **Sampling time** | **df** | **SS** | **F ratio** | ***p*** |
| --- | --- | --- | --- | --- |
|  | **One-way ANOVA on Fv/Fm** | | | |
| 120 DAP | 8 | 0.0090 | 1.1670 | 0.3352 |
| 210 DAP | 8 | 0.0307 | 2.4871 | 0.0211 |
| 300 DAP | 8 | 0.0045 | 1.3345 | 0.2443 |
|  |  |  |  |  |
|  | **One-way ANOVA on PI** | | | |
| 120 DAP | 8 | 4.6285 | 0.6261 | 0.7523 |
| 210 DAP | 8 | 5.1689 | 3.5239 | 0.0021 |
| 300 DAP | 8 | 3.4320 | 1.4487 | 0.1955 |
|  |  |  |  |  |
|  | **One-way ANOVA on LWP (MPa)** | | | |
| 120 DAP | 8 | 0.1377 | 0.8081 | 0.5981 |
| 210 DAP | 8 | 0.0681 | 0.3793 | 0.9276 |
| 300 DAP | 8 | 0.0434 | 1.1153 | 0.3662 |
|  |  |  |  |  |
|  | **One-way ANOVA on SM (% v/v)** | | | |
| 120 DAP | 8 | 25.2286 | 0.9221 | 0.5048 |
| 210 DAP | 8 | 22.6217 | 0.6511 | 0.7316 |
| 300 DAP | 8 | 64.2323 | 0.4783 | 0.8667 |
|  |  |  |  |  |

**Table S4.** Results of two-way ANOVA on below ground biomass (g), above ground biomass (g), plant height (cm) and main stem diameter (mm) at the end of Experiment 2 (greenhouse trial) to test for an inoculation treatment effect, drought effect and their interaction.

| **Source of variation** | **df** | **SS** | **F ratio** | ***p*** |
| --- | --- | --- | --- | --- |
|  | **One-way ANOVA on below ground biomass (g)** | | | |
| Inoculation | 2 | 685.56 | 0.34 | 0.7151 |
| Drought | 1 | 18593.48 | 183139.00 | <.0001 |
| Inoculation x drought | 2 | 1602.82 | 0.79 | 0.4597 |
|  |  |  |  |  |
|  | **One-way ANOVA on above ground biomass (g)** | | | |
| Inoculation | 2 | 212.23 | 0.02 | 0.9834 |
| Drought | 1 | 949483.45 | 149.86 | <.0001 |
| Inoculation x drought | 2 | 3356.60 | 0.26 | 0.7684 |
|  |  |  |  |  |
|  | **One-way ANOVA on plant height (cm)** | | | |
| Inoculation | 2 | 2196.92 | 1.32 | 0.2774 |
| Drought | 1 | 8520.22 | 10.21 | 0.0024 |
| Inoculation x drought | 2 | 798.08 | 0.48 | 0.6228 |
|  |  |  |  |  |
|  | **One-way ANOVA on main stem diameter (mm)** | | | |
| Inoculation | 2 | 0.88 | 0.30 | 0.7390 |
| Drought | 1 | 36.87 | 25.55 | <.0001 |
| Inoculation x drought | 2 | 2.55 | 0.88 | 0.4202 |
|  |  |  |  |  |

**Table S5.** Results of **a** one-way ANOVA on photosynthetic capacity (A_max_), stomatal conductance (gs_max_), water use efficiency (WUE), maximum quantum yield of PSII (Fv/Fm) and performance index (PI) of cassava to test for an inoculation treatment effect in well-watered (watered) and water-stressed (drought) plants at each sampling time in Experiment 2 (greenhouse trial). Means (± standard error) of highlighted tests are plotted in Figure 4.

| **Sampling time (DAP)** | **Watered** | | | |  | **Drought** | | | |
| --- | --- | --- | --- | --- | --- | --- | --- | --- | --- |
|  | **df** | **SS** | **F ratio** | ***p*** |  | **df** | **SS** | **F ratio** | ***p*** |
|  | **One-way ANOVA on A_max_** (μmol m^−2^ s^−1^) | | | | | | | | |
| 103 | 2 | 11.8813 | 0.8753 | 0.44 |  | 2 | 21.0360 | 1.7582 | 0.2140 |
| 109 | 2 | 3.2573 | 0.1714 | 0.84 |  | 2 | 23.9373 | 1.7066 | 0.2227 |
| 116 | 2 | 15.4173 | 2.3878 | 0.13 |  | 2 | 26.5720 | 1.1498 | 0.3493 |
| 119 | 2 | 2.0280 | 0.3859 | 0.69 |  | 2 | 5.8013 | 8.2172 | **0.0056** |
| 123 | 2 | 11.4893 | 3.1587 | 0.08 |  | 2 | 0.2773 | 0.4107 | 0.6722 |
| 130 | 2 | 0.3613 | 0.1025 | 0.90 |  | 2 | 3.4680 | 0.7736 | 0.4830 |
| 136 | 2 | 1.7613 | 0.6607 | 0.53 |  | 2 | 2.1493 | 0.7978 | 0.4728 |
| 140 | 2 | 3.2760 | 0.6069 | 0.56 |  | 2 | 0.4493 | 0.0409 | 0.9600 |
|  | **One-way ANOVA on gs_max_** (mmol m^−2^ s^−1^) | | | | | | | | |
| 103 | 2 | 510.5333 | 1.7010 | 0.22 |  | 2 | 292.1333 | 2.2553 | 0.1474 |
| 109 | 2 | 1066.1333 | 0.7407 | 0.50 |  | 2 | 396.9333 | 0.4356 | 0.6567 |
| 116 | 2 | 547.6000 | 0.9930 | 0.40 |  | 2 | 3620.9333 | 1.9021 | 0.1916 |
| 119 | 2 | 488.9053 | 1.0323 | 0.39 |  | 2 | 289.2000 | 7.7742 | **0.0068** |
| 123 | 2 | 936.4000 | 2.7514 | 0.10 |  | 2 | 250.1333 | 4.2492 | 0.0403 |
| 130 | 2 | 37.2000 | 0.2987 | 0.75 |  | 2 | 14.5333 | 0.2276 | 0.7998 |
| 136 | 2 | 80.9333 | 0.3348 | 0.72 |  | 2 | 20.9333 | 0.3489 | 0.7124 |
| 140 | 2 | 154.1333 | 0.9241 | 0.42 |  | 2 | 732.1333 | 2.1816 | 0.1556 |
|  | **One-way ANOVA on WUE** | | | | | | | | |
| 103 | 2 | 0.0322 | 1.4591 | 0.27 |  | 2 | 0.0061 | 0.4084 | 0.6736 |
| 109 | 2 | 0.0119 | 0.9818 | 0.40 |  | 2 | 0.0084 | 2.9780 | 0.0891 |
| 116 | 2 | 0.0032 | 1.1889 | 0.34 |  | 2 | 0.0784 | 7.9924 | 0.0062 |
| 119 | 2 | 0.2914 | 1.1033 | 0.36 |  | 2 | 0.0008 | 1.2915 | 0.3105 |
| 123 | 2 | 0.0120 | 1.3937 | 0.29 |  | 2 | 0.0022 | 1.0395 | 0.3834 |
| 130 | 2 | 0.0235 | 0.6471 | 0.54 |  | 2 | 0.0403 | 0.3636 | 0.7026 |
| 136 | 2 | 0.0004 | 0.0658 | 0.94 |  | 2 | 0.0042 | 1.0353 | 0.3848 |
| 140 | 2 | 0.0049 | 1.0937 | 0.37 |  | 2 | 0.0269 | 2.5512 | 0.1193 |
|  | **One-way ANOVA on Fv/Fm** | | | | | | | | |
| 103 | 2 | 0.0015 | 2.2117 | 0.15 |  | 2 | 0.0018 | 3.1465 | 0.0797 |
| 109 | 2 | 0.0003 | 0.2962 | 0.75 |  | 2 | 0.0007 | 0.8953 | 0.4341 |
| 116 | 2 | 0.0029 | 0.4703 | 0.64 |  | 2 | 0.0002 | 0.1036 | 0.9024 |
| 119 | 2 | 0.0001 | 0.7220 | 0.51 |  | 2 | 0.0036 | 1.2673 | 0.3167 |
| 123 | 2 | 0.0034 | 2.8147 | 0.10 |  | 2 | 0.0018 | 0.7590 | 0.4893 |
| 130 | 2 | 0.0052 | 0.1637 | 0.85 |  | 2 | 0.0006 | 0.2262 | 0.8009 |
| 136 | 2 | 0.0015 | 1.8241 | 0.20 |  | 2 | 0.0016 | 0.8294 | 0.4599 |
| 140 | 2 | 0.0003 | 2.7374 | 0.10 |  | 2 | 0.0012 | 0.9592 | 0.4107 |
|  | **One-way ANOVA on PI** | | | | | | | | |
| 103 | 2 | 2.6380 | 1.6628 | 0.23 |  | 2 | 3.9423 | 1.9909 | 0.1792 |
| 109 | 2 | 1.5745 | 0.5573 | 0.59 |  | 2 | 4.5508 | 1.8812 | 0.1947 |
| 116 | 2 | 5.1191 | 2.1918 | 0.15 |  | 2 | 0.7414 | 0.4406 | 0.6537 |
| 119 | 2 | 0.0135 | 0.0126 | 0.99 |  | 2 | 8.3997 | 3.9706 | **0.0475** |
| 123 | 2 | 0.8925 | 1.8538 | 0.20 |  | 2 | 0.5803 | 0.9050 | 0.4304 |
| 130 | 2 | 0.2730 | 0.3810 | 0.69 |  | 2 | 0.0883 | 0.1684 | 0.8470 |
| 136 | 2 | 1.1239 | 2.6521 | 0.11 |  | 2 | 0.2015 | 0.3129 | 0.7371 |
| 140 | 2 | 0.5642 | 1.7948 | 0.21 |  | 2 | 0.4649 | 0.9623 | 0.4096 |

**Table S6.** Results of one-way ANOVA on relative yield reduction (RYR) and drought susceptibility index (DSI) of cassava to test for an inoculation treatment effect at final sampling in Experiment 2 (greenhouse trial). Means with different letters are significantly different according to a Tukey post-hoc test (*p* < 0.05). Values highlighted in green are plotted in Figure 3.

| **df** | **SS** | **F ratio** | ***p*** | **Inoculation treatment** | **Mean (± standard error )** |
| --- | --- | --- | --- | --- | --- |
| **One-way ANOVA on relative yield reduction (RYR)** | | | | | |
| 2 | 0.1935 | 1.2138 | 0.3140 | A1 | 0.30 ± 0.09 |
|  |  |  |  | C3 | 0.30 ± 0.07 |
|  |  |  |  | Ca | 0.12 ± 0.11 |
|  |  |  |  |  |  |
| **One-way ANOVA on drought susceptibility index (DSI)** | | | | | |
| 2 | 1.4094 | 5.4448 | 0.0112 | A1 | 1.21 ± 0.12 a |
|  |  |  |  | C3 | 0.97 ± 0.08 ab |
|  |  |  |  | Ca | 0.67 ± 0.14 b |
|  |  |  |  |  |  |

**
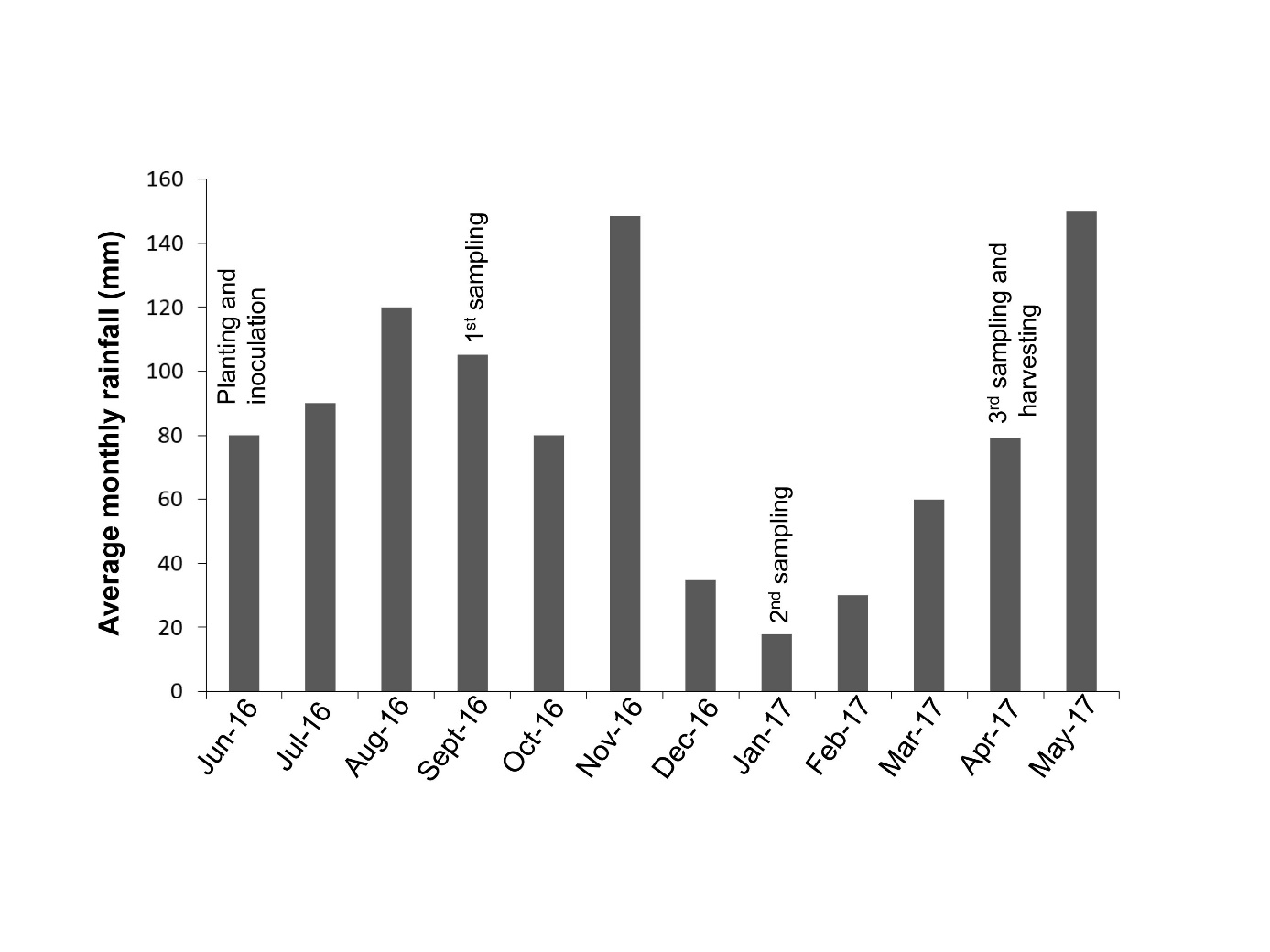
**

**Figure S1.** Monthly accumulated precipitation in Ukwala-Kawayo, Kenya for the period from June 2016 to May 2017 (field trial).


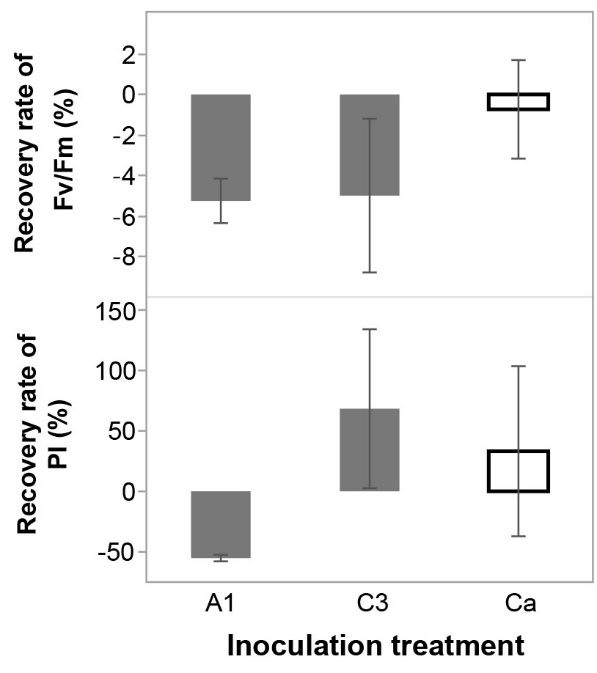


**Figure S2.** Effect of inoculation with *R. irregularis* isolates C3 and A1 on recovery rate of the maximum quantum yield of PSII (Fv/Fm) and performance index (PI) during the re-watering period in plants that had been subjected to drought in Experiment 2 (greenhouse trial). Error bars represent +1 S.E. There were no significant differences observed at *p* < 0.05.
